# Quantitative Trait Locus Mapping for Verticillium wilt Resistance in an Upland Cotton Recombinant Inbred Line Using SNP-Based High Density Genetic Map

**DOI:** 10.3389/fpls.2017.00382

**Published:** 2017-04-05

**Authors:** Koffi Kibalou Palanga, Muhammad Jamshed, Md. Harun or Rashid, Juwu Gong, Junwen Li, Muhammad Sajid Iqbal, Aiying Liu, Haihong Shang, Yuzhen Shi, Tingting Chen, Qun Ge, Zhen Zhang, Tussipkan Dilnur, Weijie Li, Pengtao Li, Wankui Gong, Youlu Yuan

**Affiliations:** State Key Laboratory of Cotton Biology, Key Laboratory of Biological and Genetic Breeding of Cotton, The Ministry of Agriculture, Institute of Cotton Research, Chinese Academy of Agricultural SciencesAnyang, China

**Keywords:** upland cotton, Verticillium wilt, disease index, disease incidence, recombinant inbred lines, quantitative trait loci

## Abstract

Verticillium wilt (VW) caused by *Verticillium dahlia* Kleb is one of the most destructive diseases of cotton. Numerous efforts have been made to improve the resistance of upland cotton against VW, with little progress achieved due to the paucity of upland cotton breeding germplasms with high level of resistance to VW. *Gossypium barbadense* was regarded as more resistant compared to upland cotton; however, it is difficult to apply the resistance from *G. barbadense* to upland cotton improvement because of the hybrid breakdown and the difficulty to fix resistant phenotype in their interspecific filial. Here we reported QTLs related to VW resistance identified in upland cotton based on 1 year experiment in greenhouse with six replications and 4 years investigations in field with two replications each year. In total, 119 QTLs of disease index (DI) and of disease incidence (DInc) were identified on 25 chromosome of cotton genome except chromosome 13 (c13). For DI, 62 QTLs explaining 3.7–12.2% of the observed phenotypic variations were detected on 24 chromosomes except c11 and c13. For DInc, 59 QTLs explaining 2.3–21.30% of the observed PV were identified on 19 chromosomes except c5, c8, c12-c13, c18-c19, and c26. Seven DI QTLs were detected to be stable in at least environments, among which six have sGK9708 alleles, while 28 DInc QTLs were detected to be stable in at least environments. Eighteen QTL clusters containing 40 QTLs were identified on 13 chromosomes (c1-c4, c6-c7, c10, c14, c17 c20-c22, and c24-c25). Most of the stable QTLs aggregated into these clusters. These QTLs and clusters identification can be an important step toward Verticillium wilt resistant gene cloning in upland cotton and provide useful information to understand the complex genetic bases of Verticillium wilt resistance.

## Introduction

Cotton is one of the most important and widely cultivated fiber crops in more than 80 countries (Jamshed et al., 2016). It is also the second most important source of edible oil and protein (Zhang et al., 2014b). Two tetraploid cotton species, *Gossypium hirsutum* L. (*G. hirsutum*) (upland cotton) and, *Gossypium barbadense* L. (*G. barbadense*) are the main cultivated worldwide. It was suggested that these tetraploid species originated from a hybridization of two diploid species, *G. arboreum* (providing A_t_ sub-genome) and *G. raimondii* (providing D_t_ sub-genome) about 1–2 million years ago (Chen et al., 2007). Verticillium wilt (VW), a cotton disease caused by the soil borne fungus *Verticillium dahliaea* Kleb is one of the most destructive disease limiting successful cotton production (Zhang et al., 2015). The disease causes substantial cotton yield losses and serious fiber quality reduction (Paplomatas et al., 1992; DeVay et al., 1997; Fang et al., 2013b).

Up to now, the most cost effective and practical way of VW management is to develop cotton cultivars harboring resistance or tolerance to the pathogen using conventional breeding and transgenic strategies (Zhang et al., 2000; Jian et al., 2003; Mert et al., 2005; Wang et al., 2014). Among the two main cultivated cotton species, *G. barbadense* is regarded as resistant while *G. hirsutum* generally susceptible to VW disease (Wilhelm et al., 1974; Fang et al., 2013a). Most of the studies have used interspecific mating systems to breed resistant upland cotton cultivars however some factors like the hybrid breakdown, weakness and sterility have hindered the application of the resistant resources from *G. barbadense* into *G. hirsutum* breeding programs (Fang et al., 2013a).

VW resistance inheritance has been reported to be under the control of dominant or partially dominant genes in studies involving early generations of interspecific populations derived from crosses between upland cotton and *G. barbadense* (Bell and Presley, 1969; Wilhelm et al., 1972, 1974; Du et al., 2004). In the intraspecific crosses of upland cotton, different inheritance patterns have been reported (Barrow, 1970; Mert et al., 2005; Cai et al., 2009; Zhang et al., 2014a). This controversies may be incurred by the different resistance sources and homozygosity of resistance genes, virulence and inoculum levels of the pathogen, evaluation methods, environmental factors (especially soil temperature and moisture), and plant maturity (Zhang et al., 2014a). Moreover, VW resistance has been reported to be under the control of recessive genes (Roberts and Staten, 1972; Devey and Roose, 1987).

VW resistant QTLs have been identified on almost all the cotton chromosomes except on c10 and c18 using mainly interspecific and very few intraspecific of upland cotton populations (Jian et al., 2003; Mert et al., 2005; Fang et al., 2013b; Wang et al., 2014; Zhang et al., 2014a, 2015). A major QTL conferring resistance and explaining 23.1–27.1% of the observed phenotypic variations (PVs) to the VW defoliating strain V991 was identified on c6 in LHB22 × Jimian11 F_2:3_ population and was confirmed in LHB22 × NNG F_2:3_ population. Furthermore, in the same study, another resistance QTL explaining 14.4% of the observed PVs, originated from the susceptible parent Jimian 11 was identified on c21 indicating that both resistant and susceptible parents may contain genes/loci that contribute positively to VW resistance (Zhang et al., 2014b). QTL clusters with high contribution rate on chromosomes D9 (c23) and D7 (c16) were identified using a cross between two upland cotton cultivars, VW resistant 60182 and the susceptible Jimian 1 (Jiang et al., 2009). In a study using a map of SSR, SNP and resistance gene analog-amplified fragment length polymorphism loci markers, 21 QTLs controlling VW resistance to the defoliating pathotype BC strain were identified on 11 chromosomes and 2 linkage groups at six different days after inoculation in two different greenhouse tests using an introgressed recombinant inbred line (RIL) population (Fang et al., 2013b). In another study using a RIL population derived from susceptible upland strain, 86-1 and resistant upland strain, Prema, 12VW resistance QTLs were identified (Ning et al., 2013); among which, one major QTL explaining 62.8% of the PVs and flanked by the markers NAU3414 and NAU2954 was identified in both greenhouse tests and field investigations on chromosome D9. However, except Fang et al. (2013b) study, all of the aforementioned studies have used a map with a low genome coverage and only one has detected a stable QTLs across two different environments (Ning et al., 2013). Recently, a illumine infinium array (cottonSNP63K) has been developed and mainly contains 45,104 putative intraspecific SNP markers of *G. hirsutum* and 17,954 putative interspecific SNP markers involving *G. hirsutum* and other cotton species. With this array, the first saturated intraspecific genetic map comprising 26 chromosomes of cotton genome was constructed from a cross between two *G. hirsutum* varieties (Hulse-Kemp et al., 2015). This achievement opens a way for the construction of a high density genetic map of cotton. The use of a genetic map with higher map resolution and better genome coverage will still be valuable for QTL mapping and might provide more useful information to better understand VW resistance mechanism in upland cotton. On the other hand, plants responses to VW infection are sensitive to other factors, such as, the virulence of the strains, the level of inoculation, and the developmental and environmental factors (Bejarano-Alcázar et al., 1997). Therefore, the evaluation of VW resistance at different stages of growth (seedlings, flowering, and mature stage) and different environments (controlled conditions in greenhouse and naturally infected conditions in open field) appears to provide more promising information for VW resistant breeding practice in cotton.

During its development and field investigations, the RIL population derived from a cross between two upland cultivars 0–153 and sGK9708 showed a wide range of distribution of responses to VW onset (from resistant to highly susceptible) and some lines presented stable resistance against VW. Based on these observations and the recessive inheritance of VW resistance in upland cotton reported by some studies (Roberts and Staten, 1972; Devey and Roose, 1987) we hypothesize that the two parents must have the resistant genes for VW disease and it will be valuable to identify these QTLs in the RIL population. In order to verify that hypothesis, the PVs of the RIL population in response to VW onset under controlled greenhouse and natural field conditions was evaluated and reported with the aims (a) to estimate the heritability of VW resistance, (b) to identify new QTLs conferring resistance to VW.

## Materials and methods

### Plant material

The mapping population consists of 196 (F_6:8_) RILs developed from a cross between two upland cultivars, 0–153 an introgressed line from *G. arboretum* and the maternal parent, sGK9708 an insect resistant cultivar. The cross was made in 2001 and a RIL population was developed by multiple cycles of selfing as described elsewhere (Sun et al., 2012; Jamshed et al., 2016; Zhang et al., 2016). Both parents showed some tolerance to VW some years while susceptible in other in natural field conditions on the experimental farm of the Institute of Cotton Research, Chinese Academy of Agricultural Sciences (ICRCAAS) located in Anyang, Henan province of China. However, some lines of their progeny showed a consistent resistance or tolerance across different years of assessment. Two varieties, Zhongzimian 2 resistant against VW and Jimian 11 susceptible to VW, were used as control during greenhouse tests.

### Greenhouse tests

For the greenhouse tests, the *V. dahliae*a defoliating pathotype V991 reported to prevail in the Yangtze and Yellow river cotton growing region (Ning et al., 2013; Zhang et al., 2014b) was used for the inoculation. The strain was first activated on potato dextrose agar medium, then the strain mass was increased by cultivation in Czapek-Dox broth placed in an IncuShaker (Haerbin Donglian Electronics, China) with a shaking speed of 150 rpm/min at a temperature of 25°C for 14 days. The conidial suspension concentration was checked using a hemacytometer and adjusted to a final concentration of 1 × 10^7^ conidia.mL^−1^ for the root inoculation (Wang et al., 2014; Zhang et al., 2014b).

The greenhouse tests were conducted at the Anyang experimental farm of ICRCAAS. From 10 to 13 July 2015, acid-delinted seeds of the 196 RILs, parents and 2 controls were planted in a complete randomized design with six replications. For each replicate, 10 acid-delinted seeds were planted in a paper cup filled with sterilized sand and vermiculate in a proportion of 6:4. After emergence, seedlings in each cup were thinned to maintain five seedlings. The replicates were arranged in plastic trays placed on greenhouse benches. At 2 leaves stage, the bottom of the paper cup were gently removed with scissors and placed in a paper tray containing 10 mL of the conidia suspension which was first filtrated through double-layer cheesecloth to separate the conidia from the mycelia. The tray/cups were placed back in the plastic trays and after the suspension was sucked dry, the plants were irrigated once a week. The greenhouse temperature was maintained to 24°C during the whole experiment (Zhang et al., 2014b).

The phenotyping of VW resistance was conducted at 15 and 30 days after inoculation (DAI) following a severity rating system from 0 to 4 based on the plant leaf disease symptoms (percentage of chlorotic and necrotic leaves; Ning et al., 2013). In this system, 0 represents healthy plant without disease symptom; 1 indicates ≤ 25% of the leaf surface exhibited disease symptoms, 2 indicates 25.1–50.0% of the leaf surface exhibited disease symptoms, 3 indicates 50.1–75.0% of the leaf surface exhibited disease symptoms, and 4 indicates >75.0% of the leaf surface exhibited disease symptoms, with plants completely defoliated or dead.

For each replicate, two disease parameters were used. The disease index (DI) was calculated according to the formula:

$$\begin{array}{l} \text{DI}=\left[\Sigma \left(\text{Ni}\times \text{i}\right)/\left(\text{N}\times 4\right)\right]\times 100 \end{array}$$

Where i is the disease grade between 0 and 4, Ni, the number of plants with corresponding disease grade and N the number of plants investigated for each RIL. The disease incidence (DInc) was calculated as the ratio between the number of infected plants and the total number of plants (Zhang et al., 2015).

### Field investigations

The field investigation was conducted on the experimental farm of ICRCAAS in Anyang, Henan Province. Phenotypic data were collected during the mature stage in 2009 (AY09), 2013 (AY13), 2014 (AY14). In 2015, two sets of phenotypic data were taken, at flowering stage (AYF15) and maturity stage (AYM15), respectively. The field for the investigations was highly infected in natural conditions by a mixture of isolates *V. dahliae*. The experimental design was a complete randomized design with two replicates and seeds were sown in a single row plots following the local recommendations for crop management. The rows were 5 m long and 0.8 m apart. The seedlings in each row were thinned to 25 at two leaf stage. DI and DInc were calculated using the formulae described above.

### Statistical analysis of phenotypic data

Phenotypic data collected for the two disease parameters in different environments and plant stages of growth were considered and analyzed as separate traits. Taken together, 14 different traits were recorded for the RIL population, parents and controls in both greenhouse tests and field investigations. The traits means were calculated using SPSS 17.0 (SPSS, Chicago, Illinois, US) and the correlation between the different traits were estimated using the Spearman's rank correlation coefficient. A one-way analysis of variance (ANOVA) of each parameter was conducted using the statistical package SAS version 9.1 and the least significant difference (LSD) was used to compare the treatment means. The broad-sense heritability (H^2^) was estimated using the formula defined by H^2^ = Var(G)/Var(P) (Khan et al., 2010), Where Var(G) and Var(P) are the genotypic and the phenotypic variance.

### The linkage map used for QTLs identification

A high-density genetic map constructed with single nucleotide polymorphisms (SNPs) based on CottonSNP70K chip (Zhang et al., 2017) and simple sequence repeats (SSRs; Sun et al., 2012) was used for the QTLs identification (Supplementary Table 1). The SNPs and SSRs were screened and excluded after several steps based on the following criteria: the SNPs that did not show three clearly defined clusters (AA, BB, and AB); second, the SNPs that their genotypes in one or both of the parents showed heterozygosis; third, the SNPs that had no polymorphism between parents; fourth, the SNPs and SSRs for which genotyping data were missing in more than 40% of the 196 RILs; and finally, the SNPs and SSRs with a significant segregation distortion (*P* < 0.001). The map spanned a total distance of 2,856.73 cM with 26 linkage groups, all of which were allocated into chromosomes of cotton genome, and an average marker interval of 1.20 cM. The quality criteria assessments of the genetic linkage map indicated that the segregation distortion of mapped markers was low (34.78% of the mapped markers showed distorted segregation) and that the collinearity of SNP and SSR markers between the genetic and physical maps had a sufficient consistency with their locations on both maps across the whole genome (Zhang et al., 2017).

### QTLs mapping

Windows QTL Cartographer 2.5 (Wang et al., 2011) was used to identify the QTLs by Composite Interval Mapping method (CIM) using a strict threshold through 1000 permutation test and a walking speed of 1 cM. QTL identification was done separately for each trait in different environments. Positive additive effect means that the favorable alleles come from the parent 0 to 153 while a negative additive effect indicates that the favorable alleles come from the parent sGK9708. The percentage of the observed PV explained by a QTL was estimated at the highest probability peak. The QTL nomenclature was as followed: the QTL designations begins with “q” followed by the trait abbreviation, the chromosome number and the QTL serial number (Sun et al., 2012; Jamshed et al., 2016). QTLs with fully or partially overlapped confidence intervals detected for the same parameters in two or more environments were considered as same QTL and declared as stable one.

## Results

### Phenotypic DI and DInc values of the parents and controls in greenhouse tests

**Figure 1:**
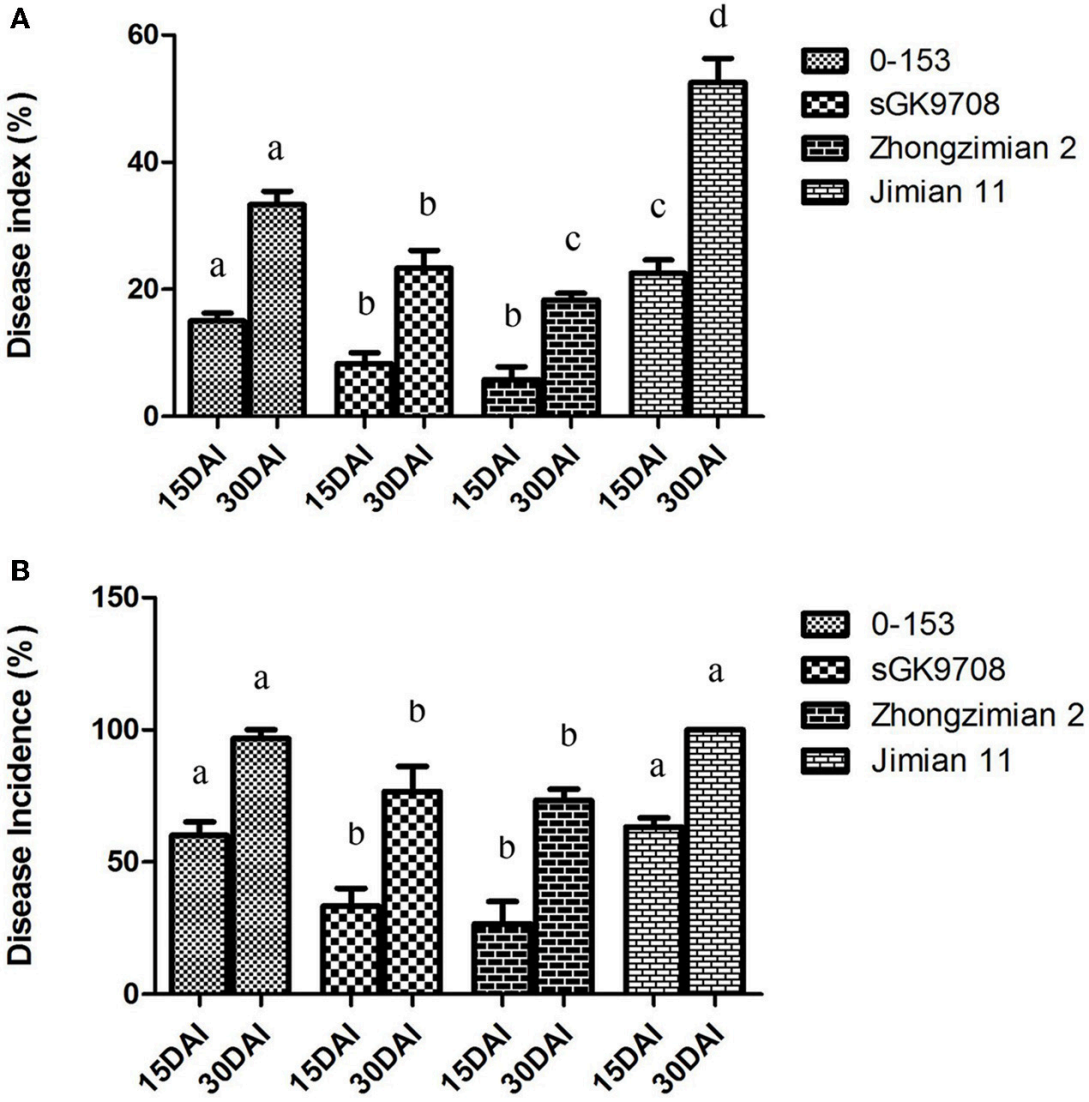
**Verticillium wilt DI (A)** and DInc **(B)** of the two parents (0–153 and sGK9708), the resistant Zhongzimian 2 and the susceptible Jimian 11. Data were collected at 15 and 30 DAI. The error bars show the standard deviation. a, b, c indicate the significances in LSD tests in 15 and 30 DAI, respectively.

At 15 DAI, the highest DI value was recorded in the susceptible Jimian 11 (22.5%), followed by 0–153 (15%) and sGK9708 (8.33%), while the lowest DI value was recorded in the resistant cultivar Zhongzimian 2(6.67%). LSD test at *P* = 0.05 revealed no significant difference between the DI values of the parent sGK9708 and the control Zhongzimian 2. However, these two values were significantly lower than those of 0–153 and Jimian 11. Furthermore, the DI value of 0–153 was significantly lower than that of Jimian 11 (Figure 1A). The highest DInc value was recorded in Jimian 11 (63.33%), followed by 0–153 (60%) and sGK9708 (33.33%), while the lowest DInc value was recorded in Zhongzimian 2 (26.68%). Susceptible Jimian 11 and 0–153 had similar DInc values, which were significantly higher than those of sGK9708 and Zhongzimian 2. No significant difference of DInc was observed between sGK9708 and Zhongzimian 2 (Figure 1B).

At 30 DAI, a similar trend was observed for DI values. Jimian 11 also had the highest DI value (52.50%) while Zhongzimian 2 had the lowest (18.53%). The parent 0–153 had the second highest DI value (33.48%) while sGK9708 had a DI value of 23.33%. LSD test revealed a significant difference of DI values between sGK9708 and Zhongzimian 2. Both of them were significantly lower than those of 0–153 and Jimian 11, while the latter two did not showed any significant differences (Figure 1A). Jimian 11 and 0–153 had the highest DInc values (100 and 96.67%, respectively) with no significant difference observed according to the LSD test. The DInc values of the remaining two varieties Zhongzimian 2 and sGK9708 were 73.33 and 76.67%, respectively, with no significant differences observed between them, but they were significantly lower than those of the susceptible Jimian 11 and 0–153 (Figure 1B).

### Phenotypic DI and DInc values of the RILs in greenhouse tests

**Figure 2:**
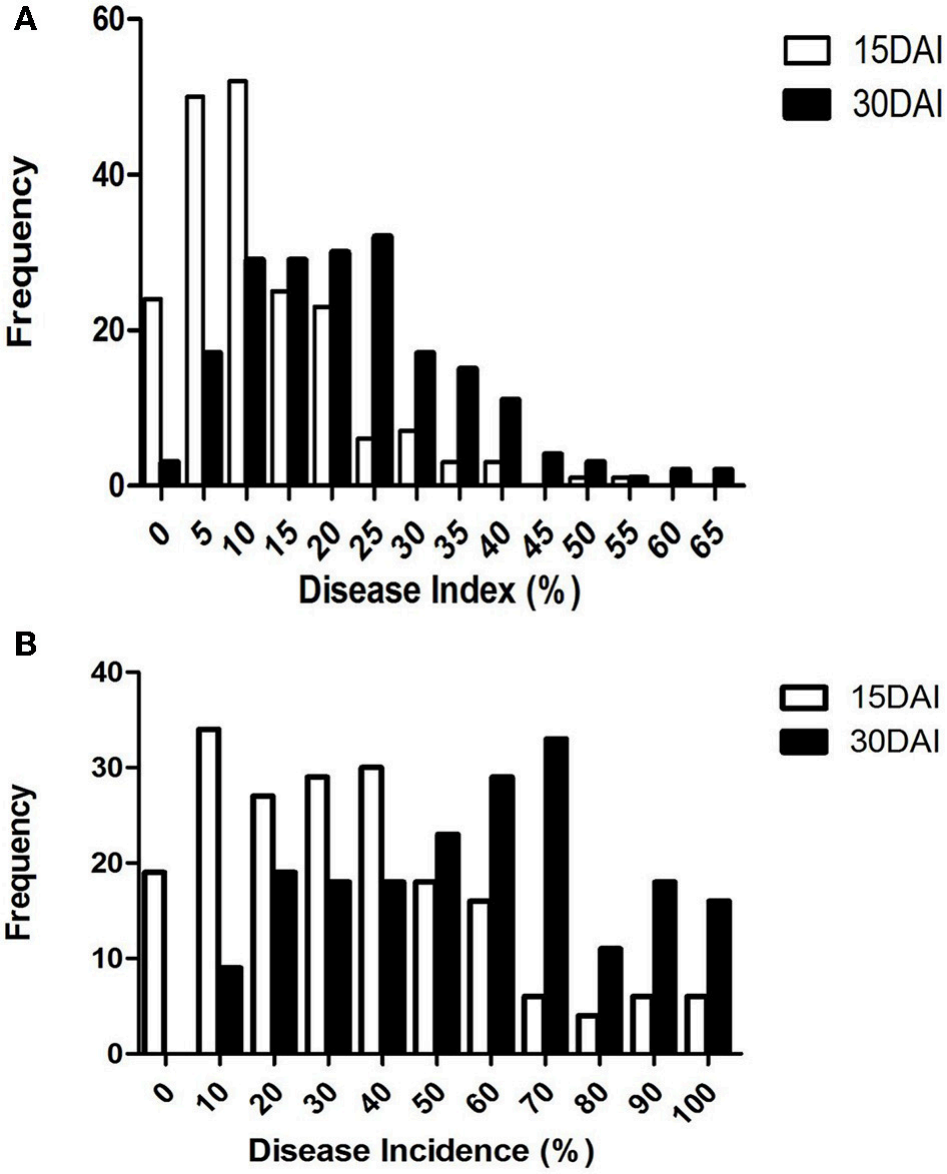
**Distribution of DI (A)** and DInc **(B)** in the RIL sGK9708 × 0–153 population. Data were collected at 15 and 30 DAI.

At 15 DAI, the DI values of the RIL population varied from 0.00 to 53.63% while the DInc values varied from 0.00 to 100%. At 30 DAI, the DI values varied from 1.25 to 62.50% while the DInc values varied from 5 to 100% (Table 1, Figure 2). The broad-sense heritability varied from 0.45 at 30 DAI to 0.66 at 15 DAI for DI and from 0.44 at 15 DAI to 0.50 at 30 DAI for DInc (Table 1, Figure 2). The mean DI value of the RIL population at 15 DAI was 11.60%, which was close to that of the mid-parent. The mean DI value of the RIL population at 30 DAI was 21.90% which was close to that of the resistant parent sGK9708 (23.33%). In the case of DInc the mean DInc values of the RILs at two DAIs were both close to those of the resistant parent sGK9708 (Table 1, Figure 2). Relatively strong correlations were observed between the two parameters at the two different DAIs with the Spearman's rank correlation coefficient (Table 2). The strongest (r_sp_ = 0.96) correlation was observed between DI and DInc values at 15 DAI, followed by the correlation between DI and DInc values at 30 DAI (Table 2).

**Table 1:** **Descriptive statistics and broad-sense heritability (H^**2**^) of Disease Index and Disease Incidence in greenhouse tests and field investigations**.

**Test**	**Phenotype**	**Env**	**RIL population**						**Parent**			**H^2^**
			**Mean**	**Min**	**Max**	***SD***	**Skewness**	**Kurtosis**	**0–153**	**sGK9708**	**Midparent**	
Greenhouse	Disease index (%)	15 DAI	11.60	0.00	52.63	9.53	0.75	0.51	15	8.33	11.67	0.66
		30 DAI	21.90	1.25	62.50	12.60	0.87	0.77	33.48	23.33	28.41	0.45
	Disease incidence (%)	15 DAI	33.71	0.00	100	25.42	0.80	0.10	60	33.33	46.67	0.44
		30 DAI	57.35	5.00	100	25.61	−0.10	−0.71	96.67	76.67	86.67	0.50
FIELD	Disease index (%)	AY09	55.86	27.93	76.00	9.15	−0.14	−0.79	61.85	41.95	51.90	0.42
		AY13	30.32	4.00	68.00	12.79	0.40	−0.15	33.33	46.05	39.69	0.39
		AY14	51.25	17.00	83.00	11.58	−0.29	0.42	57.39	55.51	56.45	0.45
		AYF15	17.96	1.00	47.00	9.52	0.65	0.17	21.61	11.40	16.50	0.12
		AYM15	57.87	30.00	81.00	8.94	−0.50	0.19	62.77	77.86	70.00	0.33
	Disease incidence (%)	AY09	96.38	77.00	100	4.42	−1.45	2.12	95.24	90.91	93.07	0.14
		AY13	62.96	17.00	100	17.66	0.37	−0.29	77.78	89.47	83.63	0.37
		AY14	75.08	32.00	100	14.15	0.70	0.27	76.92	77.50	77.21	0.49
		AYF15	33.31	3.00	84.00	16.25	0.52	0.04	42.70	17.50	30.10	0.28
		AYM15	82.13	53.00	100	10.89	−0.19	−0.51	97.05	100	98.53	0.47

**Table 2:** **Spearman's rank correlation coefficients among the 14 traits in greenhouse tests and field investigations**.

**Traits**	**15 DAI DI**	**30 DAI DI**	**AY09 DI**	**AY13 DI**	**AY14 DI**	**AYF15 DI**	**AYM15 DI**	**15 DAI DInc**	**30 DAI DInc**	**AY09 DInc**	**AY13 DInc**	**AY14 DInc**	**AYF15 DInc**
30 DAI DI	0.690^**^												
AY09 DI	ns	ns											
AY13 DI	ns	ns	0.291^**^										
AY14 DI	0.175^*^	0.217^*^	0.623^**^	0.220^**^									
AYF15 DI	0.161^*^	ns	0.163^*^	0.146^*^	0.311^**^								
AYM15 DI	ns	ns	0.466^**^	ns	0.423^**^	ns							
15 DAI DInc	0.715^**^	0.958^**^	ns	ns	0.207^*^	ns	ns						
30 DAI DInc	0.891^**^	0.698^**^	ns	ns	ns	ns	ns	0.765^**^					
AY09 DInc	ns	ns	0.534^**^	0.187^**^	0.225^**^	ns	ns	ns	ns				
AY13 DInc	ns	ns	0.156^*^	0.840^**^	ns	ns	ns	ns	ns	0.168^*^			
AY14 DInc	ns	0.141^*^	0.281^**^	ns	0.854^**^	0.356^**^	ns	ns	ns	ns	ns		
AYF15 DInc	ns	ns	ns	ns	0.352^**^	0.900^**^	ns	ns	ns	ns	ns	0.423^**^	
AYM15 DInc	ns	ns	0.224^**^	ns	ns	ns	0.786^**^	ns	ns	ns	ns	ns	−0.158^*^

* *Indicates a significant level at p < 0.05*,

** *Indicates a significant level at p < 0.01 and ns is the absence of significance*.

### Phenotypic evaluations of the parents and the RILs in field investigations

In the field investigations, the DI values of 0–153 varied from 21.61% in AYF15 to 62.77% in AYM15 while the DI values of sGK9708 varied from 11.40% in AYF15 to 77.86% in AYM15. DInc values of 0–153 and sGK9708 varied from 42.70% and 17.50% in AYF15 to 97.05 and 100% in AYM15, respectively. No significant differences between the two parents for DI and DInc values in all of the years and stage of measurements except in 2013 (Table 1).

RIL population showed more susceptibility in field investigations compared to greenhouse tests. The mean values of DI in the RILs varied from 17.96% in AYF15 to 57.87% in AYM15, respectively. Similarly, the mean values of DInc in the RILs varied from 33.31% in AYF15 to 96.38% in AY09 (Table 1). For all the years, large variations were observed in the response of the RILs to VW disease onset with lines displaying more resistance or more susceptibility compared to the two parents. In AY09, AY14, and AYM15, the negative value of the DI skewness indicated a transgressive segregation toward high DI value, while in AY13 and AYF15 the positive skewness indicated a transgressive segregation toward lower DI value. For DInc, according to the skewness value, a transgressive segregation toward low DInc value was observedin AY13, AY14, and AYF15 while a transgressive segregation toward high DInc value was observed in AY09 and AYM15. The comparison of the DI and DInc values between AYF15 and AYM15 showed a drastic increase of the disease impact over the time in the field investigations as well as in greenhouse experiments. The DI value was strongly correlated with that of DInc in each year and stage of growth. Some correlations were also observed between the traits measured in greenhouse tests and the traits measured in field investigations. For example, a positive correlation was observed between DI in AY14 and DI at 15 DAI and 30 DAI in greenhouse, respectively (r_sp_ = 0.175 and 0.217; *p* < 0.05), and between DI at 30 DAI and DInc in AY14 (r_sp_ = 0.141; *p* < 0.05). Nevertheless, no correlation was also observed for the traits in some environments (Table 2).

The broad-sense heritability ranged from 0.12 in AYF15 to 0.45 in AY14 for DI and from 0.14 in AY09 to 0.49 in AY14 for DInc. The heritability was higher in greenhouse tests compared to the field investigations (Table 1).

### Mapping of QTLs associated with VW resistance

In total, 119 QTLs controlling the two disease parameters were identified on 25 chromosomes (c1-c26 except c13) using composite interval mapping method. Sixty QTLs were identified on A_t_ chromosomes, among which 30 controlling DI were mapped on 11 A_t_ chromosomes (except c11 and c13) and another 30 controlling DInc were mapped on 8 A_t_ chromosomes (except c5, c8, c12-c13), respectively. Fifty-nine QTLs were identified on D_t_ chromosomes, among which 32 QTLs controlling DI were mapped on 13 D_t_ chromosomes while 29 QTLs controlling DInc were mapped on 10 D_t_ chromosomes (except c18-c19 and c26), respectively. For the distribution of the QTLs detected in this study on homologous chromosomes, the homologous pair c3 and c17 similarly harbored 9 and 11 QTLs, the maximum on A_t_ sub-genome and D_t_ sub-genome, respectively. Another pair that harbored similar QTL number was c1 and c15, containing 9 and 8 QTLs each. Two pairs have a little bit fewer but almost similar number of QTLs (c6 with 5 and c25 with 2, c8 with 1 and c24 with 2). Three pairs carried the same number of QTLs (c7 and c16 with 2 QTLs each, c12 and c26 with 4 QTLs each). In the remaining homologous pairs, three pairs showed that the D_t_ counterparts carried fewer QTLs (c2 with 9 and c14 with 3; c5 with 7 and c19 with 2; c10 with 8 and c20 with 4), and four pairs showed that the D_t_ counterparts carried more QTLs (c4 with 3 and c22 with6; c9 with 2 and c23 with 6; c11 with 1 and c21 with 7; c13 with0 and c18 with 5).

All the DI QTLs explained the observed PVs from 3.7 to 12.2% (Supplementary Table 2). Seven of the DI QTLs were consistently identified in at least two environments were designated as stable ones. Among them, four QTLs, *qDI-c1-1, qDI-c15-1, qDI-c20-1*, and *qDI-c22-3* were identified both in greenhouse tests and field investigations with 4.4–4.5%, 4.3–5.5%, 4.3–6.7%, and 3.7–8.6% of the observed PV explained, respectively; the remaining three QTLs, *qDI-c5-2, qDI-c18-1*, and *qDI-c24-1* were identified only in field investigations with 5.9–6.5%, 4.5–7%, and 4.3–4.7% of the observed PV explained, respectively. Two stable DI QTLS were mapped on A_t_ sub-genome while five on D_t_ sub-genome. Five stable DI QTLs have favorable alleles from sGK9708 while two from 0 to 153 (Table 3). Two QTLs identified only in one environment on c18, *qDI-c18-2* and *qDI-c18-3* also give a high explanation to the observed PV of 10 and 12.2%, respectively. There were no common QTLs detected between the two DAIs and QTLs of different DAI do not share a common chromosome region. Most of the DI QTLs detected in this study were tightly linked to the SNP markers; however, nine of them were linked to the SSR markers (Supplementary Table 2).

**Table 3:** **Stable QTLs detected for Disease Index in sGK9708 × 0–153 RIL population**.

**QTLs**	**Stage of growth**	**ENV**	**Chr**	**Position (cM)**	**Nearest marker**	**Marker interval**	**LOD**	**Additive effect**	**PV%**
qDI-c1-1	Mature	AY09	c01	68.7	i24446Gh	TMB1931-i35065Gh	2.0	−7.6	4.4
	15 DAI	GrH	c01	71.5	i37131Gh	TMB1931-i27896Gh	2.0	−2.1	4.5
qDI-c5-2	Mature	AY09	c05	34.0	i35064Gh	i00595Gh-i20721Gh	2.5	−5.2	5.9
	Mature	AY13	c05	39.7	i20721Gh	i48326Gh-i16671Gh	2	−2.7	6.5
qDI-c15-1	15 DAI	GrH	c15	3.1	i02333Gh	i02393Gh-i02418Gh	2.1	−0.4	4.3
	Mature	AY09	c15	0.01	i02393Gh	i02393Gh-i02320Gh	2.4	−0.6	5.5
qDI-c18-1	Mature	AY14	c18	31.9	i20700Gh	i20372Gh-i45991Gh	2.1	−7.3	4.5
	Flowering	AYF15	c18	28.0	i20373Gh	i41786Gh-i13695Gh	3.0	−3.4	7.0
qDI-c20-1	15 DAI	GrH	c20	15.3	i22997Gh	i12353Gh-i12304Gh	2	2.4	4.3
	Mature	AY13	c20	25.2	i12304Gh	i12306Gh-i00501Gh	2	4.5	6.7
qDI-c22-3	Mature	AY14	c22	59.8	i17784Gh	i12657Gh-i39605Gh	2	8.3	4.0
	Flowering	AYF15	c22	61.0	i12638Gh	i52667Gb-i39605Gh	2	2.3	3.7
	30 DAI	GrH	c22	63.2	i39605Gh	i52667Gb-i12588Gh	4.1	5.9	8.6
qDI-c24-1	Mature	AY09	c24	42.4	i04062Gh	i04363Gh-i32953Gh	2	−6.3	4.3
	Mature	AY13	c24	39.1	i04364Gh	i46990Gh-i26254Gh	2.2	−6.0	4.7
	Flowering	AYF15	c24	44.4	i42436Gh	i04363Gh-i18616Gh	2.2	−3.1	4.7

All the DInc QTLs explained the observed PVs from 2.3 to 21.30% (Supplementary Table 3). Twenty-eight of the DInc QTLs were identified consistently in at least two environments were designated as stable ones. Among them, Four QTLs, *qDInc-c2-4, qDInc-c17-2, qDInc-c17-3*, and *qDInc-c21-2*, were identified both in greenhouse tests and field investigations with 6.8–11.4%, 3.8–8.8%, 3.6–6.8%, and 4–8% of the observed PV explained, respectively. In them, *qDInc-c2-4* was identified in five environments including three stages of growth (seedling, flowering and mature stage). Two stable QTLs, *qDInc-c22-1* and *qDInc-c22-3* were identified only in greenhouse tests in both DAIs with 5.8–6.4% and 3.8–5.5% of the observed PV explained, respectively. The remaining stable DInc QTLs were only identified in field investigations, among which, six DInc QTLs, *qDInc-c1-1, qDInc-c1-4, qDInc-c1-5, qDInc-c1-7, qDInc-c2-3*, and *qDInc-c3-1* were identified in two environments with overall 2.4%-13.4% of the observed PV explained. Three QTLs, *qDInc-c10-3, qDInc-c17-4*, and *qDInc-c21-4* were identified in three environments with overall 3.4–5.2% of the observed PV explained. Thirteen QTLs *qDInc-c1-6, qDInc-c2-6, qDInc-c6-1, qDInc-c6-2, qDInc-c10-2, qDInc-c10-5, qDInc-c15-1, qDInc-c16-1, qDInc-c17-1, qDInc-c21-1, qDInc-c21-3, qDInc-c23-3*, and *qDInc-c23-5* were identified in four environments with overall 2.3–9% of the observed PV explained (Table 4). The QTLs *qDInc-c1-1, qDInc-c1-2, qDInc-c1-3, qDInc-c20-1*, and *qDInc-c23-2* had major effect and explained 13.3–13.4, 18.5, 21.3, 11.3, and 11.9% of the observed PV, respectively (Supplementary Table 3). Of all the stable DInc QTLs, 14 were mapped on A_t_ sub-genome while 14 on D_t_ sub-genome. Nineteen stable DInc QTLs on c1-3, c6, c10, c15-c17, and c21-23, had favorable alleles from 0 to 153 while nine stable QTLs on c1-c2, c6, c10, c17, and c21-c22, had favorable alleles from sGK9708.

**Table 4:** **Stable QTLs detected for Disease Incidence in sGK9708 × 0–153 RIL population**.

**QTLs**	**Stage of growth**	**ENV**	**Chr**	**Position (cM)**	**Nearest marker**	**Marker interval**	**LOD**	**Additive effect**	**PV%**
qDInc-c1-1	Mature	AY09	C01	43.31	i25127Gh	i23690Gh-i25127Gh	4.5	14.5	13.4
	Mature	AY14	C01	43.31	i25127Gh	i23690Gh-i25127Gh	4.6	14.3	13.3
qDInc-c1-4	Mature	AY09	C01	54.41	i29752Gh	i47522Gh-i22108Gh	3.2	−0.7	6.0
	Mature	AY14	C01	54.41	i25838Gh	i45035Gh-i22108Gh	3.2	−0.7	6.1
qDInc-c1-5	Mature	AY09	C01	69.31	MUSS422	TMB1931-i39578Gh	3.1	7.5	5.9
	Mature	AYM15	C01	72.21	i39578Gh	MUSS422-i34716Gh	2.6	5.7	5.4
qDInc-c1-6	Mature	AY09	C01	90.81	i40130Gh	i36683Gh-i37328Gh	2.6	6.6	5.1
	Mature	AY14	C01	90.81	i40130Gh	i36683Gh-i37328Gh	2.4	6.5	4.7
	Flowering	AYF15	C01	96.01	i37328Gh	i00594Gh-i34358Gh	3.0	5.9	5.6
	Mature	AY13	C01	96.01	i37328Gh	i45926Gh-i34358Gh	3.4	6.6	7.0
qDInc-c1-7	Mature	AY09	C01	103.11	i30750Gh	i34358Gh-i21627Gh	2.6	−0.6	5.4
	Mature	AY14	C01	103.11	i30750Gh	i34358Gh-i21627Gh	2.5	−0.6	5.1
qDInc-c2-3	Mature	AY13	C02	33.51	i44014Gh	i45488Gh-i47606Gh	2.8	1.0	6.0
	Mature	AY13	C02	33.51	i44014Gh	i45488Gh-i47606Gh	2.8	1.0	6.0
qDInc-c2-4	seedling	30 DAI	C02	35.51	i38959Gh	i28802Gh-i33283Gh	3.1	−1.1	6.8
	Mature	AY09	C02	34.51	i03269Gh	i28802Gh-i33283Gh	5.8	−13.0	11.4
	Mature	AY14	C02	34.51	i03269Gh	i28802Gh-i33737Gh	5.6	−12.0	10.4
	Mature	AYM15	C02	34.51	i03269Gh	i28802Gh-i47606Gh	5.2	−12.8	9.8
	Flowering	AYF15	C02	34.51	i03269Gh	i28802Gh-i33737Gh	4.9	−12.3	9.3
qDInc-c2-6	Mature	AY09	C02	53.91	i04938Gh	i23705Gh-i04910Gh	2.3	5.9	4.6
	Mature	AY14	C02	53.91	i04938Gh	i23705Gh-i04910Gh	3.3	7.0	6.4
	Mature	AYM15	C02	53.91	i04938Gh	i23705Gh-i04910Gh	3.0	6.6	5.8
	Flowering	AYF15	C02	53.91	i04938Gh	i23705Gh-i04910Gh	3.2	7.1	6.2
qDInc-c3-1	Mature	AYM15	C03	28.11	i42939Gh	i49233Gh-i39617Gh	1.0	2.9	2.4
	Flowering	AYF15	C03	28.11	i42939Gh	i27582Gh-i15520Gh	2.9	7.1	6.6
qDInc-c6-1	Mature	AY09	C06	32.41	i36502Gh	BNL1064-i17327Gh	2.5	−6.4	5.6
	Mature	AY14	C06	32.41	i36502Gh	BNL1440-i17327Gh	2.5	−6.5	5.5
	Mature	AYM15	C06	32.41	i36502Gh	NAU1054a-i17327Gh	2.5	−6.7	5.7
	Flowering	AYF15	C06	32.41	i36502Gh	BNL1440-i17327Gh	2.9	−7.4	6.5
qDInc-c6-2	Mature	AY09	C06	44.21	i25621Gh	i25316Gh-i41095Gh	2.6	6.4	6.0
	Mature	AY14	C06	42.81	i33647Gh	i17327Gh-i41095Gh	3.1	7.5	6.9
	Mature	AYM15	C06	44.21	i25621Gh	i39487Gh-i41095Gh	2.5	6.3	5.6
	Flowering	AYF15	C06	44.21	i25621Gh	BNL1440-i17327Gh	2.7	6.7	6.0
qDInc-c10-2	Mature	AY09	C10	25.10	i00425Gh	i32110Gh-i00380Gh	3.5	7.1	4.3
	Mature	AY14	C10	25.10	i00425Gh	i32110Gh-i00380Gh	5.0	8.9	6.3
	Mature	AYM15	C10	25.10	i00425Gh	i32110Gh-i00380Gh	6.1	10.5	9.0
	Flowering	AYF15	C10	25.10	i00425Gh	i32110Gh-i00380Gh	4.4	8.5	5.5
qDInc-c10-3	Mature	AY14	C10	37.70	i25153Gh	i43465Gh-i44312Gh	3.3	−8.4	5.2
	Mature	AYM15	C10	37.70	i25153Gh	i43465Gh-i30970Gh	2.3	−6.2	4.8
qDInc-c10-5	Mature	AY09	C10	50.30	i27166Gh	i49272Gh-i46214Gh	1.4	3.1	2.3
	Mature	AY14	C10	50.30	i27166Gh	i49272Gh-i46214Gh	2.0	3.6	2.9
	Mature	AYM15	C10	50.30	i27166Gh	i49272Gh-i46214Gh	2.6	4.7	5.0
	Flowering	AYF15	C10	50.30	i27166Gh	i49272Gh-i46214Gh	2.0	3.6	2.7
qDInc-c15-1	Mature	AY09	C15	53.91	i02866Gh	MUSS045-i42159Gh	3.3	5.8	6.9
	Mature	AY14	C15	53.91	i02866Gh	MUSS045-i42159Gh	3.2	5.8	6.7
	Mature	AYM15	C15	53.91	i02866Gh	i22772Gh-i42159Gh	3.2	5.9	6.8
	Flowering	AYF15	C15	53.91	i02866Gh	MUSS045-i42159Gh	3.4	6.1	7.0
	15 DAI	GrH	C15	56.71	i02866Gh	i02759Gh-i42159Gh	1.9	−0.7	5.2
qDInc-c16-1	Mature	AY09	C16	133.11	i33420Gh	i26919Gh-i01773Gh	2.1	3.7	3.7
	Mature	AY14	C16	132.61	i29345Gh	i26919Gh-i01773Gh	2.0	3.7	3.6
	Mature	AYM15	C16	133.11	i33420Gh	i26919Gh-i01773Gh	2.0	3.7	3.6
	Flowering	AYF15	C16	133.11	i33420Gh	i26919Gh-i01773Gh	2.0	3.8	3.6
qDInc-c17-1	Mature	AY09	C17	0.01	NAU3419b	NAU3419b-i51597Gb	1.6	3.8	3.6
	Mature	AY14	C17	0.01	NAU3419b	NAU3419b-i51597Gb	1.5	3.8	3.5
	Mature	AYM15	C17	0.01	NAU3419b	NAU3419b-i51597Gb	2.0	4.1	4.1
	Flowering	AYF15	C17	0.01	NAU3419b	NAU3419b-i51597Gb	3.4	7.5	7.5
qDInc-c17-2	30 DAI	GrH	C17	3.91	i51597Gb	NAU3419b-i03091Gh	3.8	1.0	8.8
	Flowering	AYF15	C17	5.91	i03090Gh	i51597Gb-i03104Gh	2.4	5.4	5.4
	Mature	AY14	C17	6.21	i03090Gh	i51597Gb-i14800Gh	1.7	3.9	3.8
qDInc-c17-3	30 DAI	GrH	C17	9.31	i14800Gh	i03090Gh-i14804Gh	3.0	0.9	6.8
	Mature	AY09	C17	9.31	i14800Gh	i03090Gh-i14804Gh	1.6	3.8	3.6
	Mature	AYM15	C17	9.31	i14800Gh	i03090Gh-i14804Gh	1.7	4.1	4.0
qDInc-c17-4	Mature	AY09	C17	22.91	i32350Gh	i49503Gh-i25863Gh	2.0	−3.8	3.4
	Mature	AY14	C17	22.91	i32350Gh	i49503Gh-i25863Gh	2.6	−4.4	4.5
	Mature	AYM15	C17	22.91	i32350Gh	i49503Gh-i25863Gh	2.3	−4.1	4.0
qDInc-c21-1	Mature	AY09	C21	26.41	i06951Gh	i33389Gh-i06953Gh	1.9	4.6	4.3
	Mature	AY14	C21	26.41	i06951Gh	i42676Gh-i06953Gh	2.1	4.8	4.7
	Mature	AYM15	C21	26.41	i06951Gh	i33389Gh-i06953Gh	1.8	4.6	4.1
	Flowering	AYF15	C21	26.31	i06951Gh	i33389Gh-i06953Gh	1.8	5.8	4.0
qDInc-c21-2	Flowering	AYF15	C21	27.41	i06953Gh	i06951Gh-i15945Gh	3.3	−0.8	8.0
	15 DAI	GrH	C21	28.51	i06953Gh	i06951Gh-i15945Gh	1.8	−0.8	4.0
qDInc-c21-3	Mature	AY09	C21	31.91	i15953Gh	i15945Gh-i33707Gh	2.2	5.6	5.3
	Mature	AY14	C21	31.91	i15953Gh	i41270Gh-i33707Gh	2.3	5.9	5.4
	Mature	AYM15	C21	31.91	i15953Gh	i15945Gh-i33707Gh	2.1	5.6	5.0
	Flowering	AYF15	C21	31.91	i15953Gh	i41270Gh-i15953Gh	1.6	5.2	3.8
qDInc-c21-4	Mature	AY09	C21	37.71	i24354Gh	i41998Gh-i07021Gh	1.9	−5.4	4.3
	Mature	AY14	C21	37.71	i24354Gh	i41998Gh-i07021Gh	2.0	−5.6	4.5
	Mature	AYM15	C21	37.71	i24354Gh	i41998Gh-i07021Gh	2.0	−5.6	4.4
qDInc-c22-1	15 DAI	GrH	C22	38.71	i12810Gh	i12810Gh-i17838Gh	2.6	−1.7	6.4
	30 DAI	GrH	C22	42.61	i12810Gh	i12810Gh-i17838Gh	2.8	−1.2	5.8
qDInc-c22-3	15 DAI	GrH	C22	61.01	i12638Gh	i20172Gh- i12588Gh	1.7	0.9	3.8
	30 DAI	GrH	C22	63.21	i12638Gh	i20172Gh- i12588Gh	2.6	1.0	5.5
qDInc-c23-3	Mature	AY09	C23	76.61	i30317Gh	i50013Gb-i15787Gh	1.4	2.7	3.0
	Mature	AY14	C23	76.61	i30317Gh	i50013Gb-i15787Gh	1.4	2.8	3.0
	Mature	AYM15	C23	76.61	i30317Gh	i50013Gb-i15787Gh	1.5	2.8	3.1
	Mature	AY13	C23	77.61	i06315Gh	i06251Gh-i06352Gh	2.9	0.5	5.7
qDInc-c23-5	Mature	AY09	C23	86.51	i06454Gh	i40526Gh-i06458Gh	2.0	4.4	4.3
	Mature	AY14	C23	86.51	i06454Gh	i25467Gh-i06456Gh	1.8	4.3	4.0
	Mature	AYM15	C23	86.51	i06454Gh	i25467Gh-i06456Gh	1.7	4.1	3.7
	Flowering	AYF15	C23	86.51	i06454Gh	i25467Gh-i06512Gh	2.3	5.0	5.1

### Co-localization of QTLs/QTL-clusters

QTL clustering is frequently observed in plants and also observed in cotton (Lacape et al., 2010; Jamshed et al., 2016). In this study, if confidence interval of two or more QTLs of different parameters overlapped completely or partially, we declared that region as a cluster. Clustering of the two disease parameter QTLs was present on some chromosomes (c1-c4, c6-c7, c10, c14, c17, c20-c22, c24-c25 (Supplementary Table 4). Twenty-three clusters were identified in this study and most stable QTLs resided in these cluster regions. Maximum QTLs number (4) in one cluster was found on c17 and QTLs harbored in that cluster explained 3.5–8.8% of the observed PVs. Chromosome 22 contained 3 clusters explaining 3.7–8.6% of the observed PVs. Two chromosomes, c1 and c21, contained two clusters, which explained overall 4.3–21.3% of the observed PV. Eleven chromosomes including c2-c4, c6-c7, c10, c14, c17, c20, c24, and c25 contained one cluster and explained overall 2.4–11.3% of the observed PV. The presence of clusters indicated congruence of QTLs detected for the different parameters in this study. The details of each cluster are summarized in Supplementary Table 4 and Supplementary Image 1.

In some clusters, QTLs share the same genomic position within short confidence interval. For example on c15, *qDI-c15-3*, and *qDInc-c15-3* identified in AY09 shared the same position and nearest marker, which explained, respectively, 4.4 and 2.4% of the observed PV. Similar results were observed on five other chromosomes including c3, c17, c21, c22 and c24 for *qDI-c17-1* and *qDInc-c17-1, qDI-c21-2* and *qDInc-c21-4, qDI-c22-3* and *qDInc-c22-4, qDI-c24-2* and *qDInc-c24-2*, respectively (Table 5).

**Table 5:** **QTLs sharing the same genomic regions detected in sGK9708 × 0-153 RIL population**.

**QTLs**	**Growth stage**	**Env**	**Position**	**PV (%)**	**Nearest marker**	**Marker interval**
qDI-c3-6	Mature	AY14	75.51	5.1	i00715Gh	i31031Gh-i32187Gh
qDInc-c3-3	Mature	AY13	75.51	6.2	i00715Gh	i38618Gh-i32187Gh
qDI-c17-1	Mature	AY09	0.01	6.8	NAU3419b	NAU3419b-i03090Gh
qDInc-c17-1	Mature	AY09	0.01	3.6	NAU3419b	NAU3419b-i51597Gb
	Mature	AY14	0.01	3.5	NAU3419b	NAU3419b-i51597Gb
	Mature	AYM15	0.01	4.1	NAU3419b	NAU3419b-i51597Gb
	Flowering	AYF15	0.01	7.5	NAU3419b	NAU3419b-i51597Gb
qDI-c21-2	Mature	AY13	37.71	5.3	i24354Gh	i33707Gh-i00449Gh
qDInc-c21-4	Mature	AY09	37.71	4.3	i24354Gh	i41998Gh-i07021Gh
	Mature	AY14	37.71	4.5	i24354Gh	i41998Gh-i07021Gh
	Mature	AYM15	37.71	4.4	i24354Gh	i41998Gh-i07021Gh
qDI-c22-3	Flowering	AYF15	61.01	3.7	i12638Gh	i52667Gb-i39605Gh
qDInc-c22-3	15 DAI	GrH	61.01	3.8	i12638Gh	i20172Gh- i12588Gh
qDI-c24-1	Mature	AY13	39.11	4.7	i04364Gh	i46990Gh-i26254Gh
qDInc-c24-2	Mature	AY13	39.11	5.5	i04364Gh	i41205Gh-i26254Gh

## Discussion

### Experimental conditions and phenotypic evaluation

In this study, an intraspecific upland cotton RIL mapping population along with parents and controls were evaluated for VW resistance under controlled greenhouse tests and natural field investigations. The disease evaluation was based on the necrotic and chlorotic areas of leaves and the number of plants showing infection. The number of replicates (6) and the number of plants in each replicate (5) combined with the resistant control (Zhongzimian 2) and susceptible control (Jimian 11) are enough precautions to increase the accuracy of our data, and thus to reduce experimental errors. The greenhouse results indicated that sGK9708 was resistant to the strain V991, which showed congruence with our hypothesis in the beginning of our study. In the field investigations, DI values of the two parents were above 35% except in AY13 when the DI of 0–153 was lower than 35% and in AYF15 when both parents were resistant. These result variations in field investigations might be explained by the fact that in the natural field conditions, the cotton plants were subjected to the pressure of a mixture of VW strains. The quantity of soil fungi, virulence of the strains, and the developmental and environmental factors (Bejarano-Alcázar et al., 1997) might contribute to these result variations. Similar results were reported in a previous study, in which a parent reckoned susceptible during field investigations turned to be resistant in the greenhouse after root wounding for the pathogen inoculation (Fang et al., 2013a). The results also indicated that 0–153 showed a tolerance (DI < 35%); several of the progenies displayed a higher level of resistance compared to the two parents. Furthermore, we observed a high level of transgressive segregation in greenhouse tests, which was also reported in previous reports (Bolek et al., 2005; Wang et al., 2008). DI values of some lines were less than that of the resistant control Zhongzimian 2 and most of the lines showed a resistance to V991. However, as expected, an about two-fold increment of DI and DInc values over time were observed in greenhouse tests at 30 DAI compared to those of 15 DAI. The level of susceptibility was higher at 30 DAI compared to 15 DAI. In the field where the RILs were subjected to a mixture of strains, the level of susceptibility was higher compared to the greenhouse results and only a few lines showed a consistent resistance across the 4 years of study (Figure 2). This contrast with the greenhouse result can be explained by the antagonist interactions between the different VW strains present in the field and the continuous change of the environmental conditions from 1 year to another. These observations were similar to those of Wang et al. (2014) and confirmed that distinct gene(s) control the resistance to different *V. dahlia* isolates and interaction between resistance QTL or genes and fungal strains occurs. Only few weak correlations were observed between the parameters measured in greenhouse tests and field investigations. This fact and the weak coefficient correlation value can be explained by the variability of the genes expressed at different stages of growth (seedlings stage in the greenhouse, flowering and mature stage in the field). Furthermore, no significant correlation was observed between the DI of AYF15 and AYM15. This fact highlighted the gene alternation in response to VW attack during different stages of growth.

Moderate heritability were observed for the two disease parameters in the greenhouse compared to the weaker heritability values obtained in the field where the experimental errors is high due to variation in the environmental conditions and the weather from 1 year to another. The low heritability and its variability in the field investigation suggest that the phenotypic variability of these two parameters was strongly subjected to the environmental effects. A combined analysis of both results confirmed the importance of the environmental factors in VW although a genetic basis of inheritance is also important.

### Map used for QTLs identification

Plenty of genetic maps have been constructed using both interspecific (Bejarano-Alcázar et al., 1997; Mert et al., 2005; Zhang et al., 2015) and intraspecific (Jiang et al., 2009; Fang et al., 2013b; Ning et al., 2013; Zhang et al., 2014b) populations. However, most of the maps used for intraspecific cross studies have very low genome coverage (< 50%) and do not allow a genome-wide detection of QTLs with high resolution. For example, the map used by Jiang et al. (2009) covered about 25% of the tetraploid cotton genome while the map used by Zhang et al. (2012) covered a total distance of 1143.1 cM, which is about 22.58% of the allotetraploid cotton genome. In another report (Ning et al., 2013) the genetic map with 279 markers only covered about 35% of the cotton genome. The use of SNP markers and their applications to the marker assisted selection (MAS) allow to circumvent the problem of low genome coverage of intraspecific genetic map and to construct high density genetic map (Wei et al., 2014; Zhang et al., 2014b, 2016). So far, only one study (Fang et al., 2013b) has used a map covering more than 50% of the cotton genome (55.7%). This map contained 882 markers including 432 SSR, 414 SNP and 36 resistance gene analog-amplified fragment length polymorphism (RGA-RFLP). However, that genetic map still did not cover all the 26 chromosomes of allotetraploid cotton. The number of QTLs detected by the aforementioned studies varied from 2 (Zhang et al., 2014b) to 41 (Jiang et al., 2009).

The cottonSNP70K genetic map used in this study covered 2865.73 cM, about 63.7% of the allotetraploid cotton genome; a much higher coverage of the genome than those of all the previous maps used for VW QTLs mapping in upland cotton. It also featured coverage of all 26 chromosomes of upland cotton for the first time for VW QTLs mapping. One hundred forty-four QTLs were detected for VW including two diseases parameters and were distributed on all the 26 chromosomes. Usefulness of SNP for QTLs mapping have been already demonstrated in the case of reniform nematode resistance (Buyyarapu et al., 2014).

### Disease parameters QTLs distribution

More than 100 QTLs related to VW have been detected on most of the 26 tetraploid cotton chromosomes except on chromosomes c6, c10, c12, and c18 (Zhang et al., 2014a,b, 2015). In this study, VW QTLs with minor contributions to the observed PV were detected on these four chromosomes. Five QTLs (3 for DI and 2 for DInc) were identified on c6, 8 QTLs (2 for DI and 6 for DInc) on c10; four DI QTLs on c12 and Three DI QTLs on c18. A meta-analysis of QTLs related to VW resistance reported the presence of QTLs on all the chromosomes except c10 and c18 (Zhang et al., 2015). However, in this study, a cluster harboring 2 QTLs was mapped on c10 and a total of three QTLs were mapped on c18 resulting from better coverage of these chromosomes in this report. Some chromosomes carried a relatively more number of DI QTLs in this study; c5 carried 7 DI QTLs, c13 and c15 harbored 6 DI QTLs each; c17 harbored five DI QTLs while c12 harbored 4 DI QTLs. Another important result of this study is the identification of QTLs of the two different disease parameters, which shared the same genomic region on c3, c15, c17, c21, c22, and c24. Fang et al. (2013b) had reported 2 QTLs sharing the same anchoring makers and the same peak position detected on c8 and c21 for disease rating and percentage of infected leaves. These QTLs are potentially tagging causative disease resistance genes and their association with this study's clusters can be sources of useful information for future VW resistance gene cloning and the design of efficient breeding of VW resistant lines in upland cotton.

Thirty-five QTLs reported in this study were identified in at least two environments; this number is more than any other number reported by previous studies. For the seven stable DI QTLs, four of them were detected both in the greenhouse and field assays. This fact is not surprising given that the strain V991 is also one of the prevailing defoliating strain in the cotton-growing field in Anyang and that there detected a positive correlation (although week) between the results of greenhouse tests and some of the field assays. Week positive correlation between greenhouse and field tests have been also reported by Zhang et al. (2012) in a VW study using a backcross inbred line population. Among the four stable QTLs, two had sGK9708 alleles and the other two 0–153 allele. It was also reported a QTL having susceptible parent allele after root wounding infection in the greenhouse using a cross between the *G. hirsutum* cv SG 747 and the resistant Prema (Fang et al., 2013a).

Most of the QTLs detected for the two parameters have minor effects; therefore, the high level of resistance observed in greenhouse tests must be due to combinations of certain positive alleles contributed from both parents provided to some of the progeny's member, the genotype required for VW resistance.

For the strain V991, 3 VW QTLs have been previously mapped on c9, c17, and c23 using a RIL population from Prema × 86-1 (Ning et al., 2013) and 2 QTLs on c6 and c21 using a F2:3 population (Zhang et al., 2014a). In this study, VW strain V991 resistance QTLs were identified on c1, c5, c10, c14, c15, c17, c20, c22, c25 for DI and on c2-c4, c7, c10, c11, c14, c15, c17, c21, and c22 for DInc. Among these QTLs, three (*qDI-c17-3, qDI-c17-4*, and *qDInc-c17-4*) were located in the vicinity (<8 cM) of the one reported by Ning et al. on c17 (Ning et al., 2013). Two other QTLs, *qDInc-c21-1* and *qDInc-c21-2*, were located at less than 9 cM of the QTL reported by Zhang et al. (2014b). The remaining QTLs have not been reported previously in upland cotton implying that they are new QTLs related to VW, thus confirming that different germplasms might possess different QTLs for the same VW isolate.

The results suggested that both sub-genome contribute equally for disease resistance and were in agreement with previous reports (Wang et al., 2014; Zhang et al., 2015). Most of the studies have reported the difficulty to reliably identify VW QTLs because of the likely relatively low contribution of each QTL to the phenotype and the low genome coverage (Zhang et al., 2014a). In this study, the high-density SNP markers used provided us a higher genome coverage allowing higher resolution in the VW QTLs mapping. For example, the detected number of VW QTLs is higher and more stable QTLs were identified across the different environments and stages of growth in this study compared to the two other VW studies (Ning et al., 2013; Fang et al., 2013b). This fact confirmed the usefulness of a high-density SNP maps for QTLs mapping at a high-resolution and a better understanding of the genetic background of VW inheritance.

## Conclusion

In this study, a RIL population derived from a cross between two upland cotton cultivars 0–153 and sGK9708 was used to identify VW QTLs resistance in various environments (greenhouse and field) and different stages of growth (seedling, flowering and Boll development stage). The nature of population (RIL), the number of replications (6) and the presence of two controls (Zhongzimian 2 and Jimian11) in our greenhouse tests allowed us to reduce the experimental error and to check the accuracy of our data. The results showed a sharp difference in the parents responses to the disease under controlled conditions in the greenhouse where they were inoculated with one strain compared to the field where they were subjected to a mixture of strains under natural conditions. This fact combined to the low to moderate level of heritability of the two parameters in both environments confirmed the necessity to evaluate a population at different stage of growth to get more information on the genetic bases of the VW resistance. The identification of 144 QTLs detected for the two parameters on all the 26 cotton chromosomes confirmed the complex genetic bases of VW resistance already reported by previous studies. In this study, clusters were identified on 20 chromosomes and most of the stable QTLs of the two disease parameters belong to these clusters. QTLs of the two different parameters sharing the same genomic region were identified on c3, c15, c17, c21, c22, and c24 indicating a common VW resistance mechanism for the two different but highly correlated parameters. Due to a better coverage of the cotton genome, this study facilitates a higher resolution of the VW QTLs mapping, provides new clues for understanding the VW complex genetic bases in upland cotton and the practical future breeding applications.

## Author contributions

WG, YY initiated the research, WG and PK designed the experiments. PK performed the experiments, PK, TD, HR, JL, JG, MI, HS, AL, TC, QG, ZZ, WL, and PL collected the data from the field and the greenhouse, PK, MJ, and HR performed the analysis, PK and MJ drafted the manuscript, YY and WG contributed to the final editing of manuscript. All authors contributed in the interpretation of results and approved the final manuscript.

### Conflict of interest statement

The authors declare that the research was conducted in the absence of any commercial or financial relationships that could be construed as a potential conflict of interest.

## Abbreviations

VW: Verticillium wilt
DI: disease index
DInc: disease incidence
DAI: day after inoculation
ns: non-significant
Env: environment
PV: phenotypic variation
H2: broad sense heritability
LOD: logarithm of odds
QTL: quantitative trait loci
SNP: single nucleotide polymorphism.
