# Supplementary material for: Quantitative Trait Locus Mapping for Verticillium wilt Resistance in an Upland Cotton Recombinant Inbred Line Using SNP-Based High Density Genetic Map
Source: Front Plant Sci. 2017 Apr 5;8:382. doi: 10.3389/fpls.2017.00382 (PMC5380748; doi:10.3389/fpls.2017.00382)
Supplement: Supplementary file 5 [file Image1.PDF]

Supplementary image 1: Chromosomal locations of Verticilliu wilt QTLs in the SNP-chip based linkage map

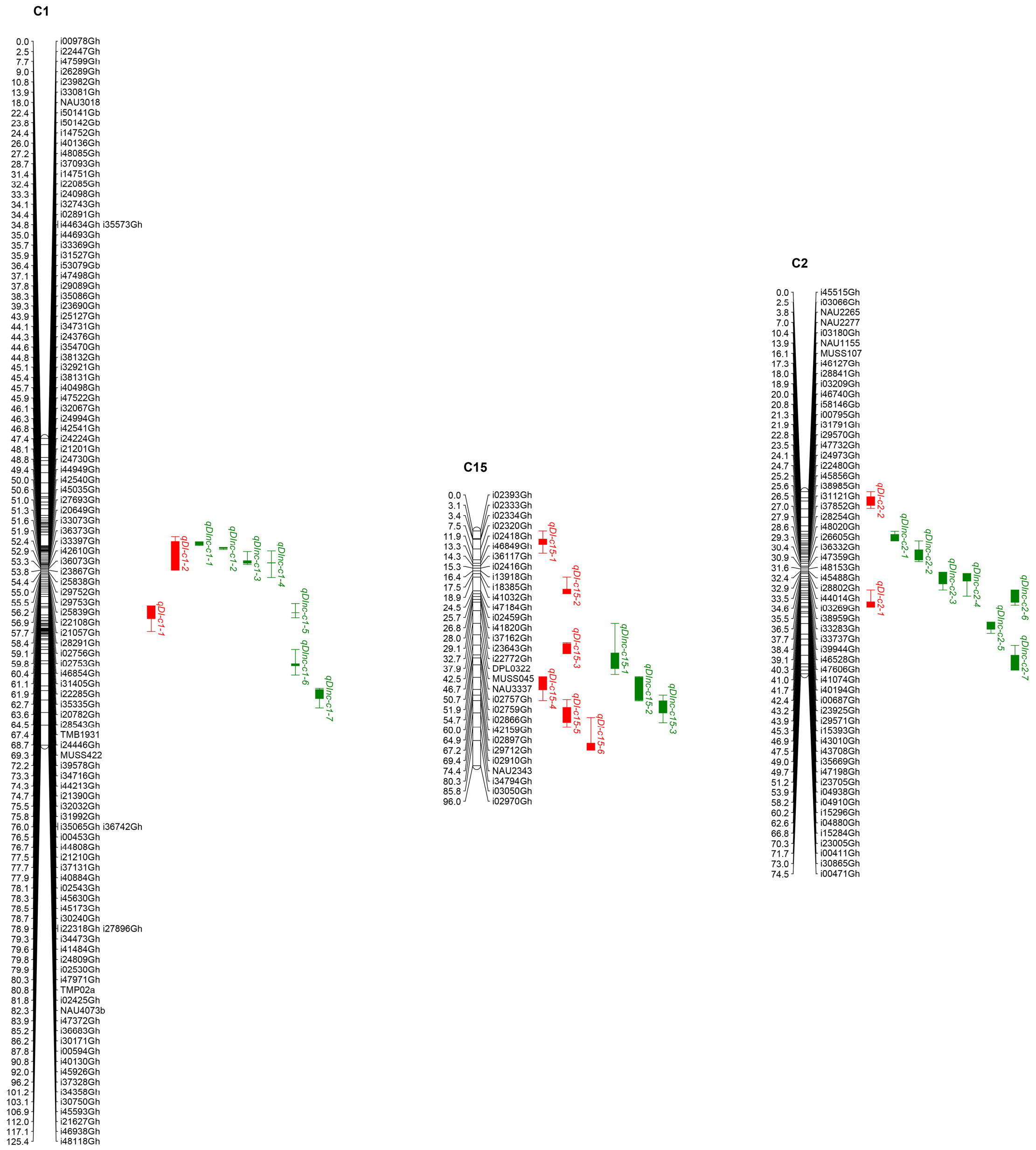

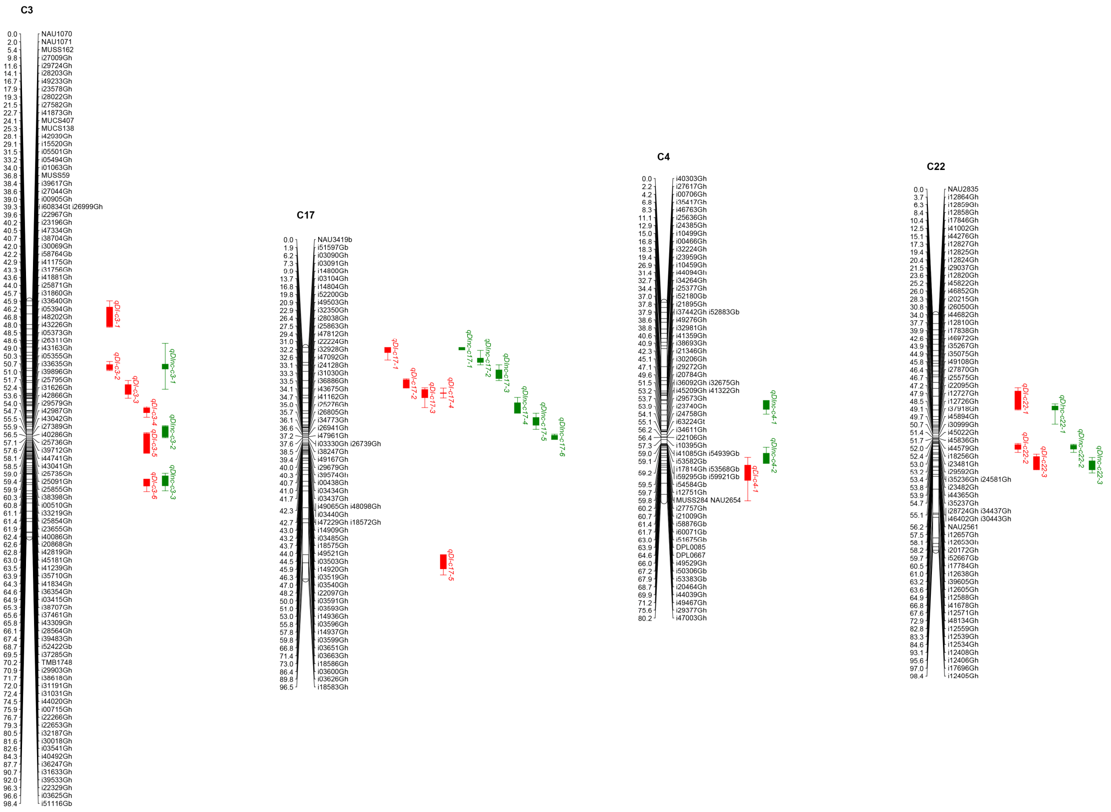
C3
C17
C4
C22

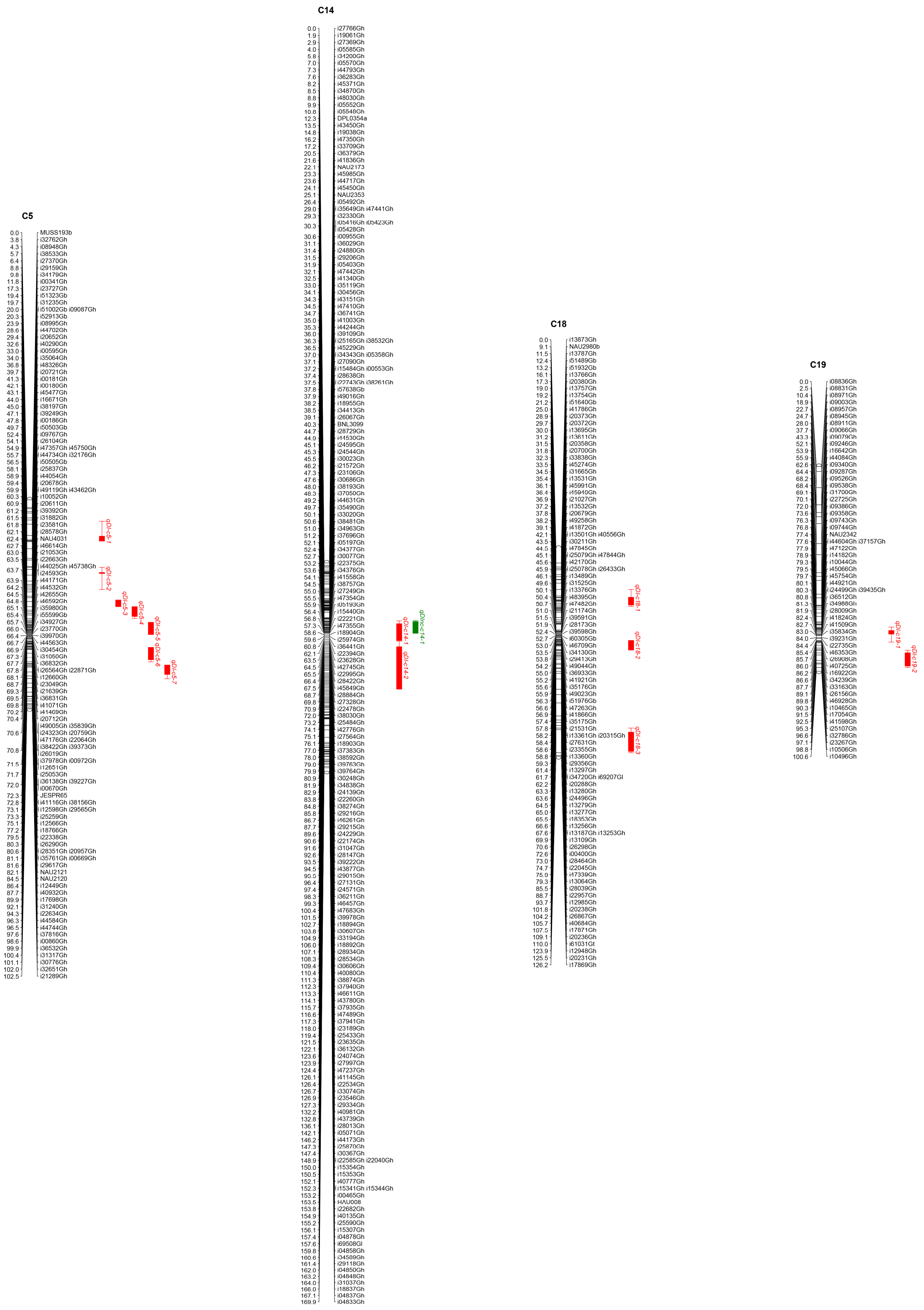
C5
C14
C18
C19

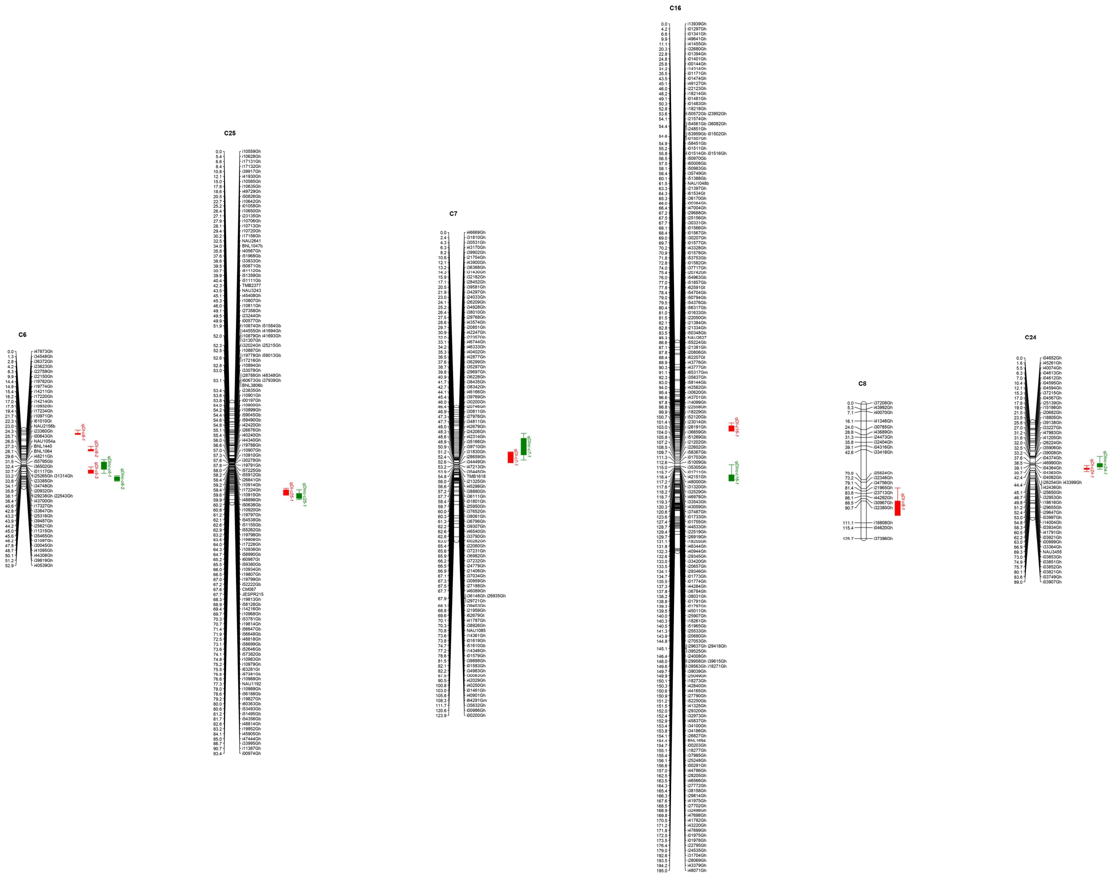
C6
C25
C7
C16
C8
C24

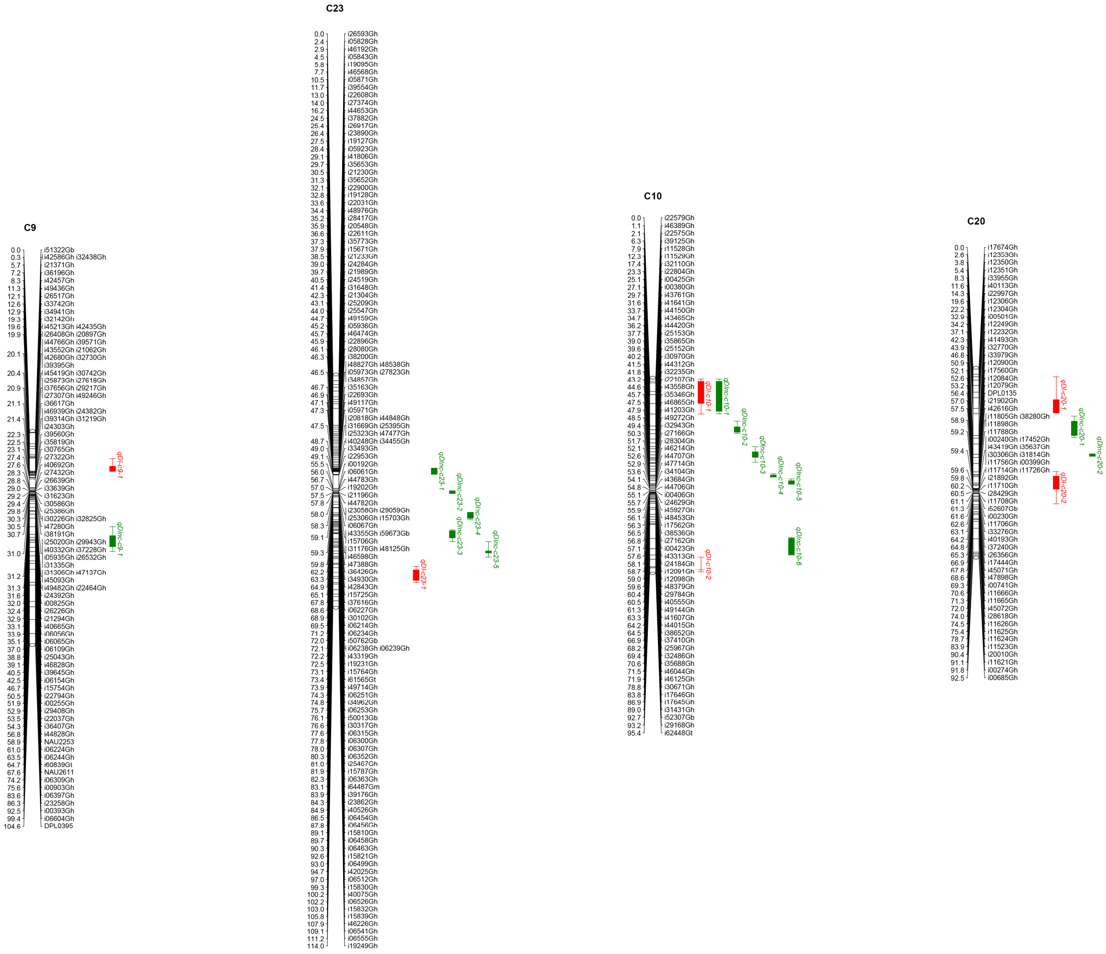
C9
C23
C10
C20

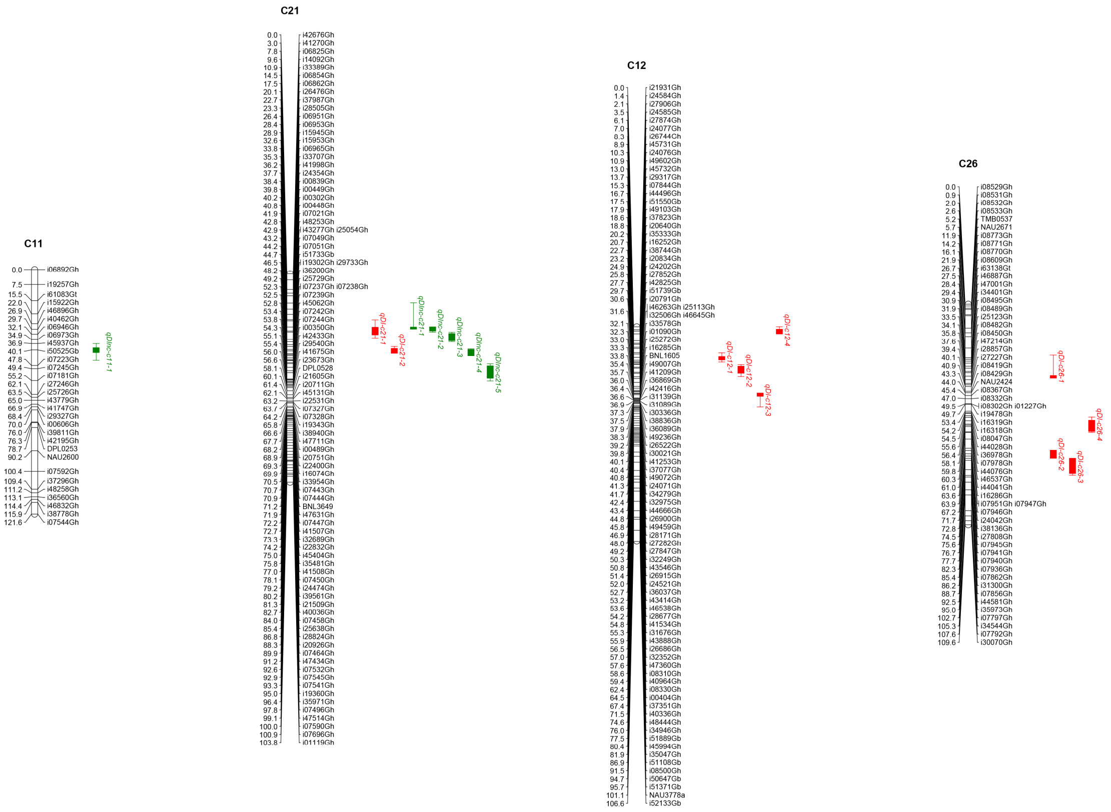
C11
qDlnc-c11-1
C21
qDl-c21-1
qDl-c21-2
qDlnc-c21-1
qDlnc-c21-2
qDlnc-c21-3
qDlnc-c21-4
qDlnc-c21-5
C12
qDl-c12-1
qDl-c12-2
qDl-c12-3
qDl-c12-4
C26
qDl-c26-1
qDl-c26-2
qDl-c26-3
qDl-c26-4
